# Clusterin knockdown has effects on intracellular and secreted von Willebrand factor in human umbilical vein endothelial cells

**DOI:** 10.1371/journal.pone.0298133

**Published:** 2024-02-16

**Authors:** Allaura A. Cox, Alice Liu, Christopher J. Ng

**Affiliations:** 1 Department of Pediatrics, University of Colorado–Anschutz Medical Campus, Aurora, CO, United States of America; 2 Department of Bioengineering, Washington University, St. Louis, MO, United States of America; Universite de Nantes, FRANCE

## Abstract

Alterations in von Willebrand factor (VWF) have an important role in human health and disease. Deficiency of VWF is associated with symptoms of bleeding and excesses of VWF are associated with thrombotic outcomes. Understanding the mechanisms that drive VWF regulation can lead to a better understanding of modulation of VWF levels in humans. We identified clusterin (*CLU*) as a potential candidate regulator of VWF based on a single cell RNA sequencing (scRNA-seq) analysis in control endothelial cells (ECs) and von Willebrand disease (VWD) endothelial colony-forming-cells (ECFCs). We found that patients with deficiencies of VWF (von Willebrand disease, VWD) had decreased *CLU* expression and ECs with low *VWF* expression also had low *CLU* expression. Based on these findings, we sought to evaluate the role of *CLU* in the regulation of VWF, specifically as it relates to VWD. As *CLU* is primarily thought to be a golgi protein involved in protein chaperoning, we hypothesized that knockdown of *CLU* would lead to decreases in VWF and alterations in Weibel-Palade bodies (WPBs). We used both siRNA- and CRISPR-Cas9-based approaches to modulate *CLU* in human umbilical vein endothelial cells (HUVECs) and evaluated VWF protein levels, *VWF* mRNA copy number, and WPB quantity and size. We demonstrated that siRNA-based knockdown of *CLU* resulted in decreases in VWF content in cellular lysates and supernatants, but no significant change in WPB quantity or size. A CRISPR-Cas9-based knockdown of *CLU* demonstrated similar findings of decreases in intracellular VWF content but no significant change in WPB quantity or size. Our data suggests that *CLU* knockdown is associated with decreases in cellular VWF content but does not affect *VWF* mRNA levels or WPB quantity or size.

## Introduction

Von Willebrand factor (VWF) is a plasma glycoprotein essential in hemostasis. VWF is primarily synthesized by endothelial cells (ECs) and released into the plasma via constitutive and regulated pathways [1, 2]. Deficiencies of VWF, along with clinical bleeding symptoms, define von Willebrand disease [3]. Excesses of VWF have been correlated with thrombotic outcomes such as myocardial infarction, stroke, and venous thromboembolism (VTE) [4–6]. Given the role that VWF plays in pathologic hemostasis and thrombosis, understanding the modulation of this problem is highly relevant.

VWF has a normal range of approximately 50–150 IU/dL [7]. While it can be acutely increased due to situations such as inflammation or infection, the wide distribution of VWF levels in the normal population suggests that there is a high degree of physiological regulation of the plasma levels of VWF and past investigations have sought to better understand the mechanisms that regulate plasma VWF levels. Known modifiers of VWF plasma levels include the ABO blood group, clearance-related mechanisms, and other possible modifiers identified on genome-wide associated studies (GWAS) [8–13]. Our analyses for potential VWF modifiers have focused on the study of intra-cellular or endothelial-based mechanisms of VWF regulation. As ECs are thought to synthesize approximately 80% of circulating VWF, they are a natural cellular source to investigate intra-cellular VWF regulation [1]. We have previously published on our experience using VWD endothelial colony forming cells (ECFCs), which are isolated from peripheral blood from individuals with VWD [14]. Previously, we screened for potential candidates for VWF regulation via a single-cell RNA-sequencing (scRNA-seq) analysis where we identified highly differentially expressed genes in control and VWD ECs. We focused on candidates that were strongly differentially expressed or had putative roles in VWF or endothelial biology [15]. Clusterin, also known as apolipoprotein J, was one such candidate identified by our screen.

Clusterin is a dimeric protein found in many different tissue types, including ECs [16]. One of clusterin’s putative roles is as a molecular chaperone with important functions in protein homeostasis [15]. As a protein that is highly associated with the secretory pathway, such as the endoplasmic reticulum/Golgi apparatus, we hypothesized that clusterin could have an important role in the regulation of VWF and it has been recently identified as a protein associated with WPBs in proximity proteomics [17]. As part of its secretory pathway, VWF is considered to be a Golgi-based protein that is then packaged into intracellular organelles called Weibel-Palade bodies (WPBs). Some of these WPBs are then trafficked to the plasma membrane for eventual release into the plasma. The regulation of this secretory pathway and its effects on VWF biology have been widely established and may play a role in VWD.

In this report, we evaluate the role of clusterin on VWF expression at the transcriptional, protein, and intracellular level in ECs.

## Materials and methods

### Materials

See S1-S4 Tables in S1 File regarding reagents.

### Generation of CLU^KO^

HUVEC cells at passage 3 were co-nucleofected with two RNPs targeting CLU. RNPs were generated by complexing Cas9-NLS (IDT) with Hs.Cas9.CLU.1.AB (CATCAAGCTGCGGACGATGC) or Hs.Cas9.CLU.1.AE (ACTCCCTGCTGGAGAACGAC) gRNA. 5ul RNP was mixed with 2x10^5^ cells and resuspended in Lonza P5 Primary Cell 4D-Nucleofector™ solution and nucleofected using CA-167 pulse. HUVEC edited pool and single cell clones at passage 4 were screened using end-point PCR using primers CLU-KO-F: TCTGGATGAATGGTGACCGC and CLU-KO-R: AATCGAGATGACACCCGCTG and agarose gel electrophoresis. After editing, cells were expanded post confirmation of knockdown. No CRISPR/Cas9 knockout CLU cell lines were used for experimentation beyond passage 7. CLU^*KO*^ cells protein and mRNA samples were harvested at confluence and then reseeded for a total of 3 consecutive passages for further protein/mRNA extraction.

### Cell lines

Human umbilical vein cells (HUVECs) were purchased from Lonza (Portsmouth, NH). All endothelial cells used in experiments described here were passaged less than 6 times. For each experiment, cells were maintained in EBM2-MV (Lonza) with supplemented FBS to 10%. Human ECFCs were isolated from healthy controls and individuals with VWD (termed “Low VWF Levels” in the previous publication and termed “VWD” in this publication to align with new diagnostic criteria) via an IRB-approved protocol with written consent from patients or legal guardians (COMIRB 15–1072, study opened 09/01/2015 and is ongoing). Details regarding ECFC isolation are noted as previous [14].

### scRNA-seq analysis

Our scRNA-seq analysis has been previously published [14]. In brief, human umbilical vein endothelial cells (HUVECs) and ECFCs isolated from healthy controls and individuals with VWD were analyzed via scRNA-seq on a 10X Genomics platform. After sequencing, bioinformatic analyses was used to generate a list of differentially expressed genes between control and VWD samples.

### siRNA transfection

siRNA transfection was conducted similar to previous publication [14]. In brief, confluent HUVECs were incubated for 24hr at 37C and transfected with RNAiMax-based transfection protocols. The final concentration of siRNAs was 1 nM. Post transfection, cells were incubated for 4 days at 37C. After 4 days, mRNA and protein were isolated.

### WPB analyses

WPB analyses were conducted similar to previous [18]. Cells were seeded on gelatin coated cover slips (Neuvitro GG121.5GELATIN). For siRNA-knockdown, cells were seeded at 60,000 cells/cm^2^, incubated for 4 days, fixed and stained. For CLU^*KO*^, then cells were seeded at 60,000 cells/cm^2^ and allowed to adhere for 24hr before fixing and staining. After fixation and permeabilization, cells were stained with a conjugated sheep anti-human Von Willebrand Factor labeled with FITC (1:1000, BioRad, AHP062F) and clusterin-α Antibody (B-5) antibody (1:1000, Santa Cruz sc-5289) and a Goat anti-mouse IgG (H+L) Secondary Cy5 antibody (1:10,000, Novus NB7602) for 1 hour at room temperature. The slides were then mounted using ProLong Glass Antifade Mounting with NucBlue Stain (Invitrogen P36983).

### VWF ELISA analysis

VWF ELISA analysis conducted similar to previous [14, 19]. In brief, cellular lysates and supernatants were collected and analyzed via established VWF ELISA analyses. VWF content is corrected by either (1) dividing by average VWF content in control samples or (2) dividing by average VWF content in control samples and total protein concentration.

### qPCR assays

After relevant experimentation, RNA was extracted from cells using miRNeasy Mini Kit (Qiagen 217084). RNA was quantified using the Invitrogen Qubit RNA BR Assay Kit (Invitrogen Q10210). RNA was then converted to cDNA using the Applied Biosystems High-Capacity cDNA Kit (TaqMan MultiScribe). After reverse transcription, 100ng of DNA was combined with Applied Biosystems TaqMan Universal PCR Master Mix plus respective Taqman probes (see S3 Table in S1 File) and run in triplicate in an Applied Biosystems StepOnePlus Real-Time PCR System machine.

### Western blot assay

Whole cells were lysed using RIPA buffer with Halt Protease and Phosphatase Inhibitor Cocktail (ThermoFisher Scientific Prod# 186128) and protein concentration was measured by Pierce^TM^ BCA Protein Assay Kit (ThermoScientific 23225). 50ug of protein was loaded into a 4–15% gradient gel (Bio Rad Mini-PROTEAN TGX Precast Gels). After electrophoresis, proteins were transferred from gel to a nitrocellulose membrane (BioRad 1620112) using the TE22 Mighty Small Transfer Tank (Hoefer) apparatus. The electroblotting was carried out at 90V for 50 minutes at 4C. After transfer, the nitrocellulose membrane was incubated with 5% milk (Bio Rad Blotting-Grade Blocker nonfat dry milk) for 1 hour at room temperature. The nitrocellulose membrane was washed for 10 minutes, three times with 1X TBST and subjected to immunoblotting with clusterin-α Antibody (B-5) HRP antibody (1:200, Santa Cruz sc-5289 HRP) overnight at 4C. The nitrocellulose membrane was washed for 10 minutes, three times with 1X TBST and visualized with Western Lightning ECL Pro (Perkin Elmer). The membrane was then incubated in Anti-beta Actin antibody - Loading Control (HRP) (ABCAM ab20272, 1:1000) for 30 minutes at room temperature. The nitrocellulose membrane was then washed for 10 minutes, three times with 1 X TBST and visualized with Western Lightning ECL Pro (Perkin Elmer).

## Results

### Decreased *CLU* expression in VWD ECFCs and in Low-VWF expressing ECs

**Fig 1:**
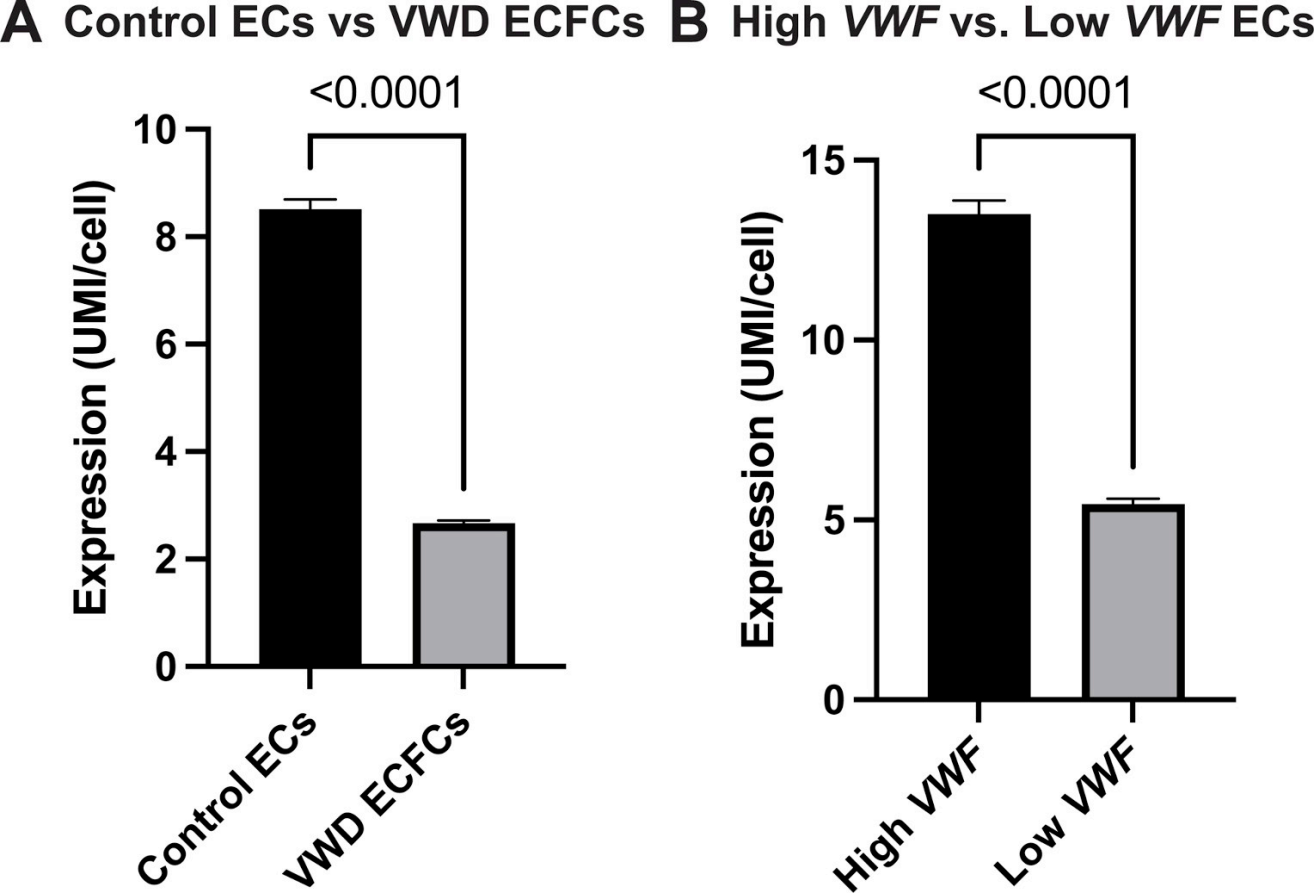
Decreased *CLU* expression in VWD ECFCs and in Low-VWF expressing ECs. *CLU* expression from control ECs and VWD ECFCs demonstrates decreased *CLU* expression in VWD ECFCs as compared to control ECs (1A). In addition, a comparison of *CLU* expression in “High VWF expressing” and “Low VWF expressing” control ECs demonstrates decreased *CLU* expression in “Low VWF expressing” ECs (1B). *CLU* expression is shown in average unique molecular identifiers (UMI)/cell. UMI is the output from the 10X Genomic platform that represents gene counts. Analyses conducted via unpaired t-test and p-values are as shown.

Based on our previous evaluation of differential expressed genes in an analysis of scRNA-seq of control and Low VWF ECs [14], *CLU* was identified as a possible candidate regulator of VWF. When comparing control ECs (n = 4153 cells) and VWD ECs (n = 6453 cells), there was a decrease in *CLU* expression in the individuals with VWD (Fig 1A). When comparing high *VWF*-expressing ECs (defined as *VWF* expression higher than or equal to the mean, n = 1581 cells) with low *VWF*-expressing cells (n = 2572 cells) from only control endothelial cell lines, there was decreased *CLU* expression in Low *VWF*-expressing ECs (Fig 1B). These findings suggested to us that *CLU* expression, at the transcriptional or protein level, was associated with a clinical VWD phenotype or with a “Low *VWF* expressing” cellular phenotype.

### siRNA-based knockdown of *CLU* results in decreased VWF protein expression but no change in WPB quantification or VWF mRNA

**Fig 2:**
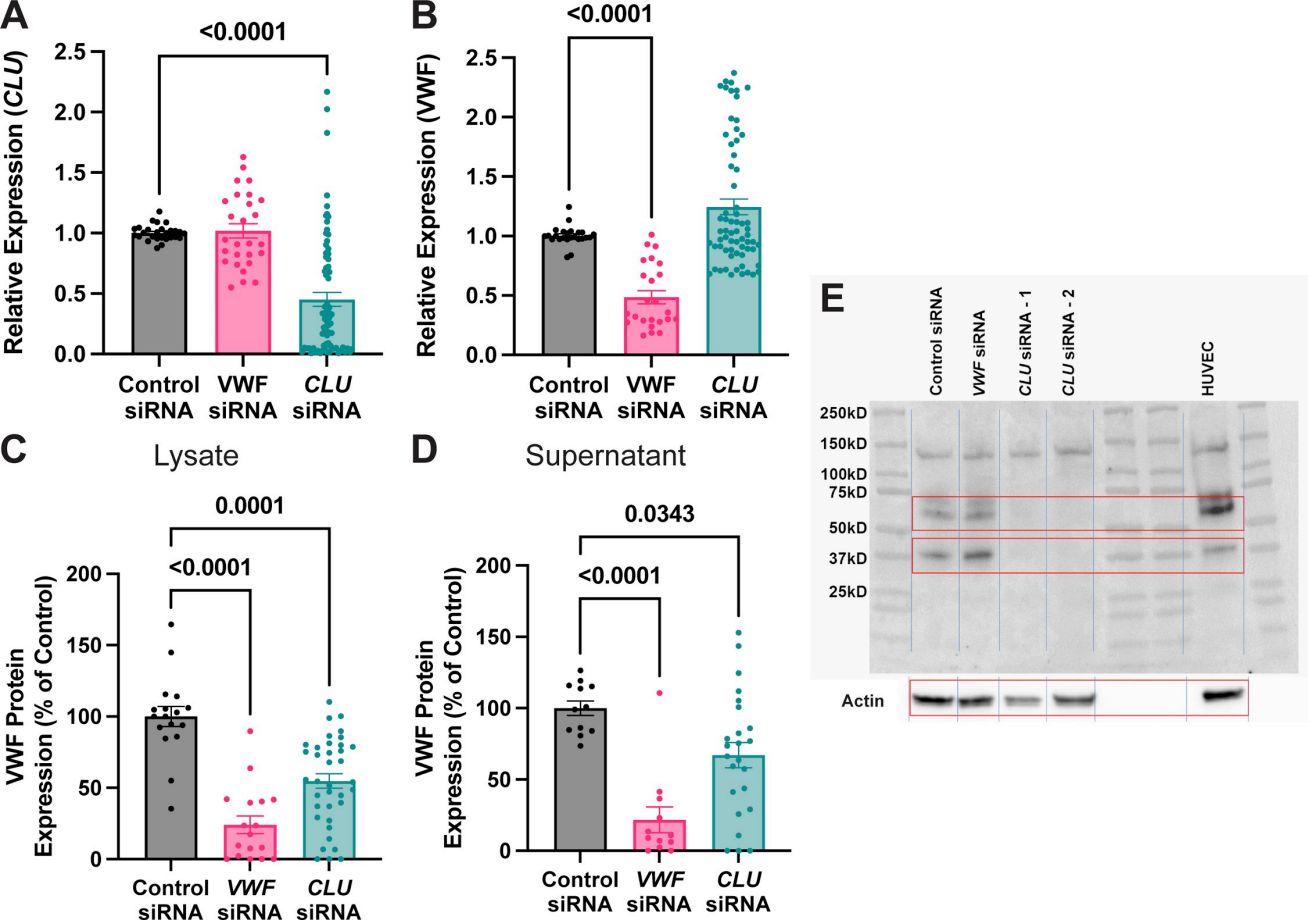
siRNA-based knockdown of *CLU* results in decreased VWF and CLU protein expression but no change in *VWF* mRNA content. qPCR analysis of *CLU* expression demonstrates significant decrease in *CLU* expression after *CLU* siRNA knockdown (2A). As expected, VWF siRNA knockdown has no effect on *CLU* expression (2A). qPCR analysis of *VWF* expression demonstrates no significant decrease in *VWF* expression in *CLU* siRNA-transfected cells but expected *VWF* knockdown in *VWF* siRNA-transfected cells as compared to control siRNA (2B). Regarding *VWF* protein content, ELISA analysis of cellular lysates after siRNA transfection demonstrates decreased VWF lysate (2C) and supernatant (2D) content in *CLU* siRNA-transfected cells. As a positive control, similar decreases in VWF content in cellular lysates and supernatants are seen after *VWF* siRNA-transfection. A western blot after *CLU* siRNA transfection demonstrates significant *CLU* knockdown with two independent *CLU* siRNAs (2E). The red boxes demonstrate expected molecular size for *CLU* (top panel) and actin (lower panel). Statistical analysis conducted via 1-way ANOVA with multiple comparison correction. P-values are as shown. Number of biological replicates ≥ 3 for represented experiments.

Based on these findings, we evaluated an siRNA-based knockdown model of *CLU* in HUVECs to determine its effects on VWF. After siRNA knockdown, we evaluated multiple parameters of CLU and VWF. We confirmed siRNA-based knockdown of our target, *CLU* via qPCR (Fig 2A). Despite significant knockdown of *CLU*, there is no significant effect on *VWF* mRNA levels (Fig 2B) suggesting that *CLU* does not regulate *VWF* in a transcriptional manner. At the protein level, we demonstrated decreased VWF protein content in cellular lysates and cellular supernatants from *CLU* knockdown HUVECs (Fig 2C and 2D). Western blot of *CLU* demonstrates knockdown of *CLU* (Fig 2E).

**Fig 3:**
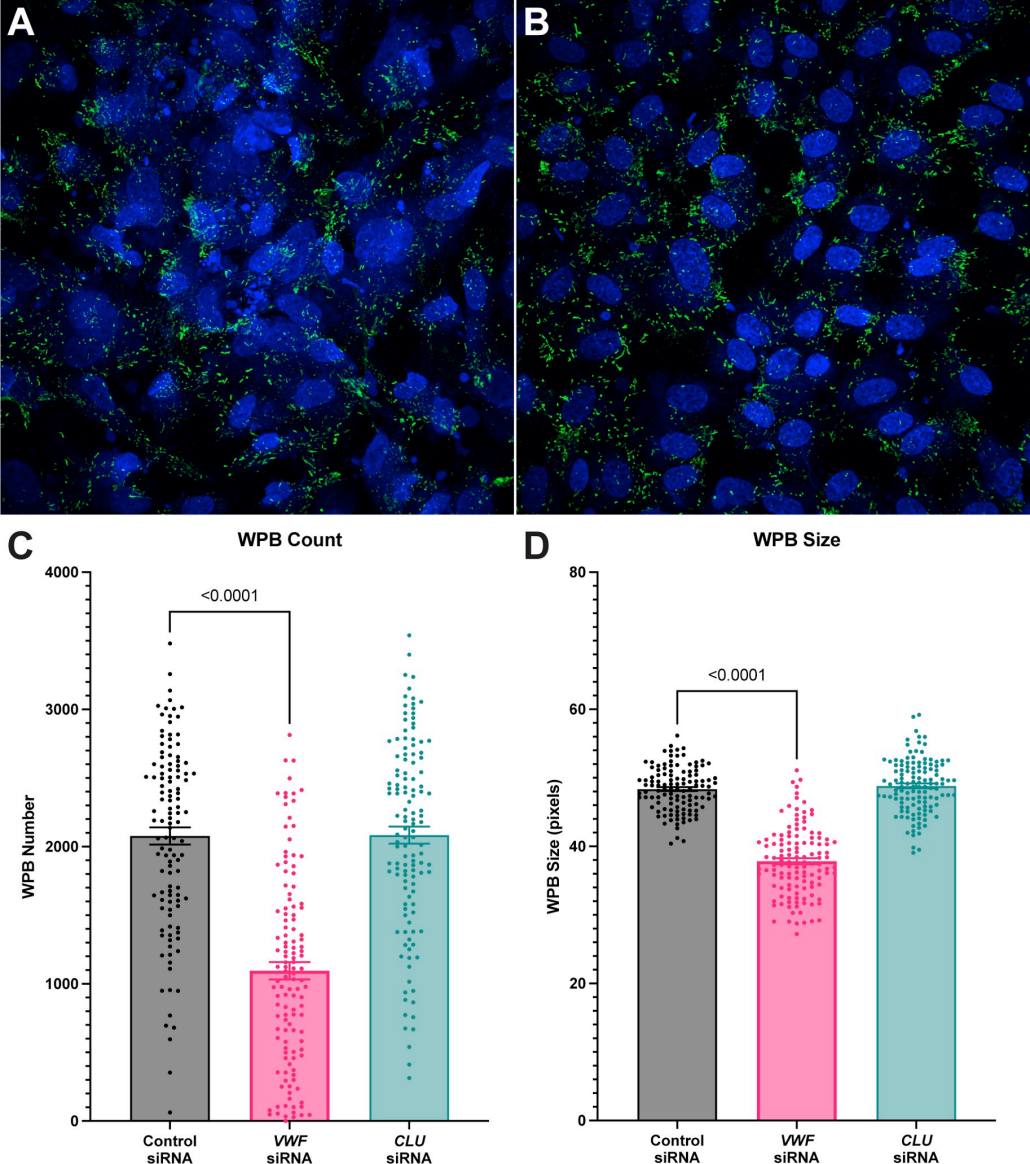
*CLU* siRNA knockdown does not change WPB number or size. *CLU* or *VWF* siRNA-treated HUVECs are analyzed for WPB content. Representative immunofluorescence images showing DAPI (blue) and VWF staining (green) from control siRNA treated (3A) and *CLU* siRNA treated cells (3B) demonstrated similar levels of confluence and the appearance of rod-like WPBs. Total number of WPBs/image care analyzed and demonstrate no significant difference between *CLU* siRNA-treated HUVECs and control siRNA-treated HUVECs. As expected, *VWF* siRNA-treated HUVECs demonstrated decreases in WPB number (3C). As previous reports have demonstrated that alterations in WPB size can also be associated with VWF abnormalities, we also analyzed the average size of WPBs after *CLU* or *VWF* siRNA treatment. There was no significant difference in WPB size when comparing *CLU* siRNA-treated HUVECs as compared to control siRNA treatment. As expected, *VWF* siRNA-treated HUVECs demonstrated decreased WPB size (3D). Statistical analysis conducted via 1-way ANOVA with multiple comparison correction. P-values are as shown. Number of biological replicates ≥ 5 for represented experiments.

As there was a significant decrease in VWF protein content, we next sought to determine if there were significant changes in WPB quantification. As alternations in VWF content have been associated with changes in WPB number or size, we analyzed both parameters in *CLU* siRNA-silenced HUVECs [20, 21]. Our analysis of WPB quantification revealed no significant difference in the number of WPBs or the size of WPBs (Fig 3C and 3D), which contrasts with our overall observation of decreased intracellular VWF content.

### CRISPR-Cas9 knockout of *CLU* results in decreased VWF protein expression but no change in WPB quantification or *VWF* mRNA levels

**Fig 4:**
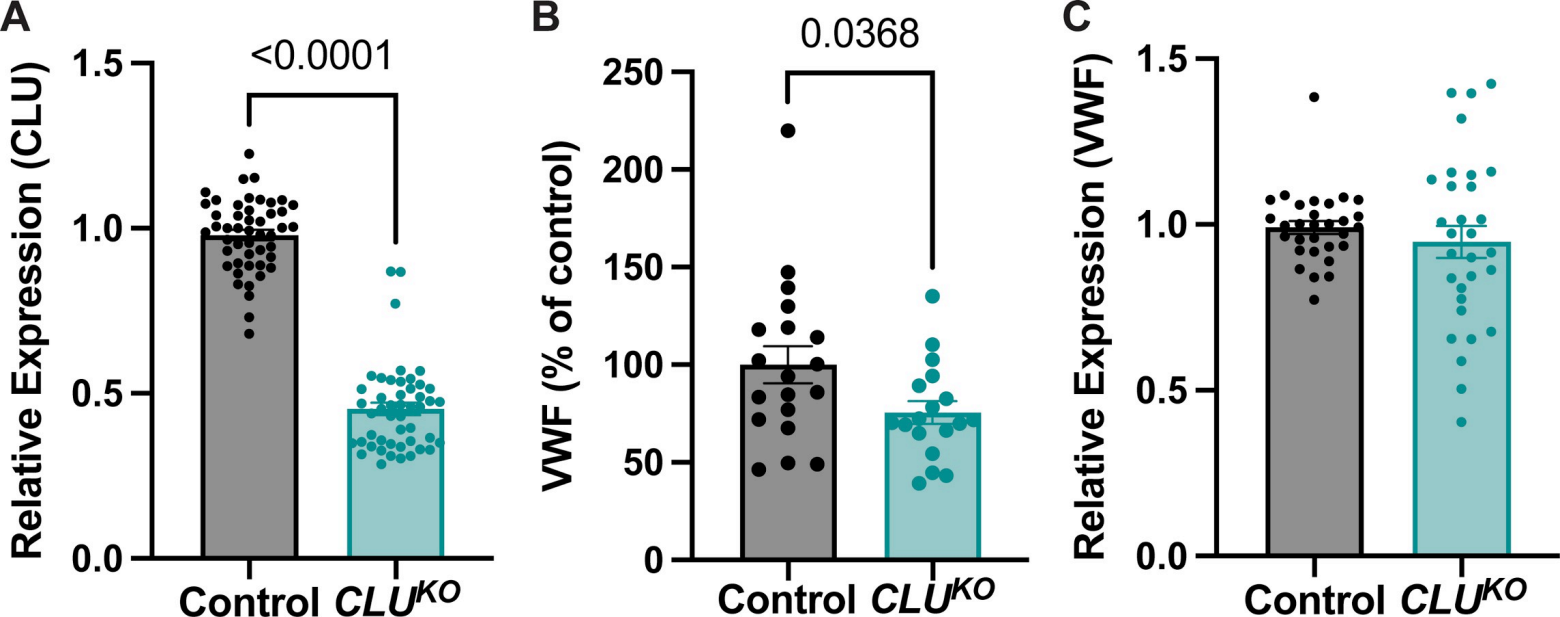
CRISPR-Cas9 knockout of *CLU* results in decreased VWF protein expression VWF mRNA. A CRISPR-Cas9 knockout HUVEC cell line (*CLU*^*KO*^) demonstrated significant decreases in *CLU* expression as measured by PCR compared to a mock/control CRISPR-Cas9 HUVEC cell line (2A). Similar to our siRNA-treated cells, the *CLU*^*KO*^ cell line demonstrated decreased VWF protein content in cellular lysates as measured by VWF ELISA (2B). Similar to our siRNA-treated cells, the *CLU*^*KO*^ cell line did not demonstrate differences in *VWF* expression (2C). For qPCR analysis, statistical analysis conducted via unpaired t-test. For VWF ELISA, statistical analyses conducted via unpaired t-test after unbiased removal of outliers via ROUT analysis with Q = 1%. P-values are as shown. Number of biological replicates ≥ 3 for represented experiments.

**Fig 5:**
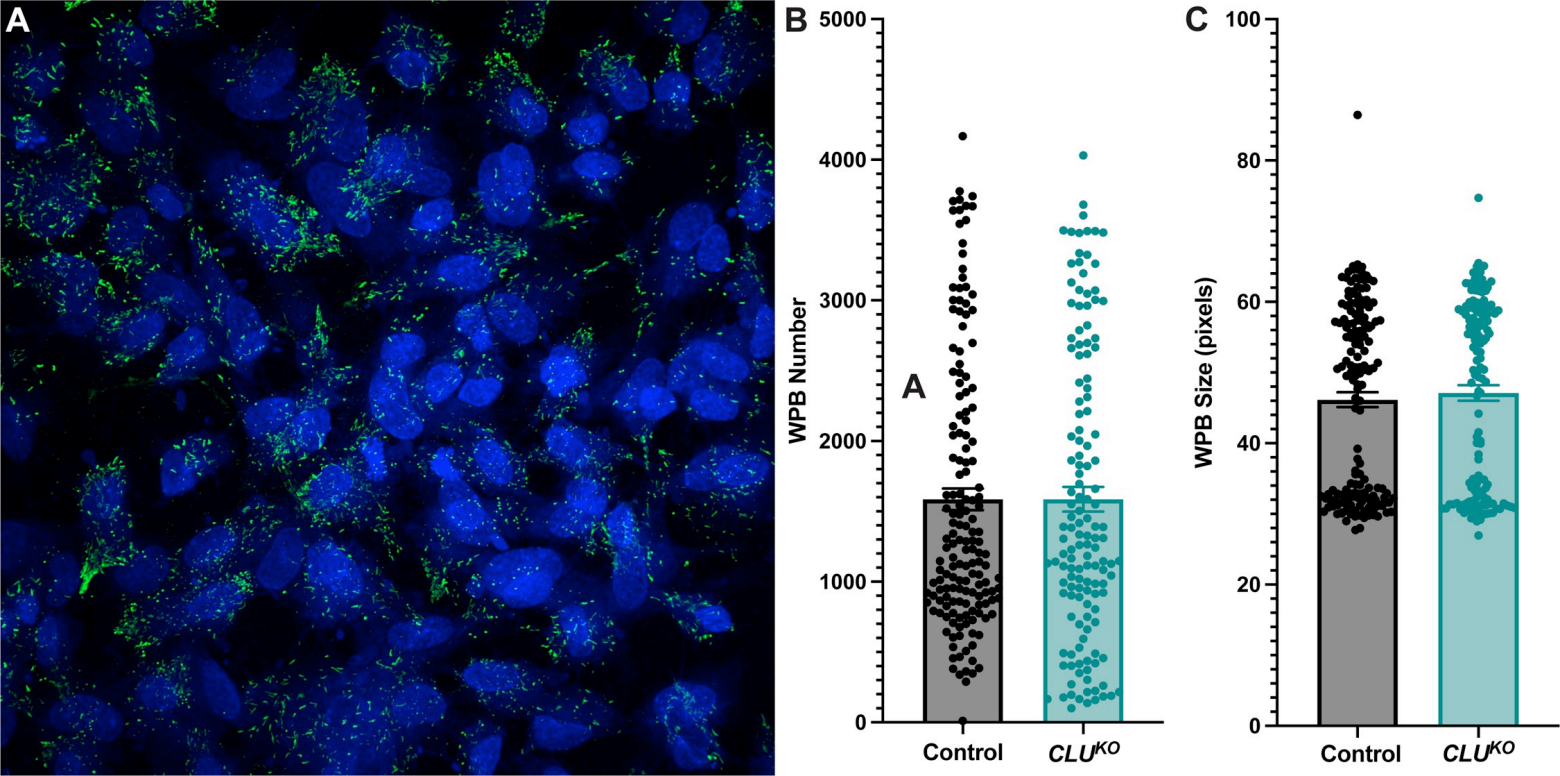
CRISPR-Cas9 knockout of *CLU* does not affect WPB number or size. A *CLU*^*KO*^ HUVEC cell line generated via CRISPR-Cas9 was analyzed for WPB number and WPB size. Representative immunofluorescent images showing DAPI (blue) and VWF staining (green) (5A) demonstrates appropriate cellular confluence and the expected shape of WPBs. Similar to our siRNA-treated HUVECs, CRISPR-Cas9 knockout of *CLU* did not demonstrate significant changes in WPB number (5B) or WPB size (5C) when compared to a control CRISPR-Cas9 HUVECs. The data did not pass normality testing (D’Agostino & Pearson test) and thus was analyzed via Mann-Whitney U test. Number of biological replicates ≥ 3 for represented experiments.

In addition to our transient siRNA-based *CLU* silencing, we created a stable knockout of *CLU* via CRISPR-Cas9 to further study the effects of *CLU* knockdown on VWF biology. After creation of this *CLU*^*KO*^, we conducted similar experiments as in our siRNA-based knockdown. *CLU* knockdown was confirmed via qPCR (Fig 4A) and similar to our siRNA experiments, intracellular protein analysis via VWF ELISA of *CLU*^KO^ HUVECs did demonstrate decreased intracellular VWF content as compared to control-knockout HUVECs (Fig 4B) but CRISPR-Cas9 *CLU*^KO^ did not demonstrate significant knockdown of *VWF* at the mRNA level (Fig 4C). Also similar to our previous experiments, we saw no significant decrease in WPB quantification or WPB size when comparing control-knockout HUVECs and *CLU*^KO^ HUVECs (Fig 5B and 5C). In summary, our experiments with a *CLU*^KO^ HUVEC cell line demonstrated similar results as with our transient *CLU* siRNA experiments.

## Discussion

In this report, we identified clusterin as a candidate regulator of VWF levels in ECs based on our previously described scRNA-seq experiments. Based on the significant differences in *CLU* gene expression in VWD ECFCs and Low-VWF expressing ECs and recent data suggest roles of *CLU* in endothelial biology and VWF [17, 22], we sought to further evaluate how clusterin may affect VWF in ECs.

Our results suggest that clusterin may influence VWF homeostasis. Knockdown of *CLU* at the mRNA level via siRNA or via CRISPR-Cas9 led to decreases in VWF content in ECs and the cellular supernatant. The decreased amount of VWF in the cellular supernatant may be secondary to the decreased VWF levels intracellularly, as decreases in the cellular supernatant were of the same magnitude as that of the cellular lysate. Interestingly, the decrease in lysate and supernatant VWF content did not correlate with a significant change in WPB quantification or size, potentially indicating that *CLU* knockdown/knockout had little effect on the WPB-based packaging/synthesis pathway. This may suggest that *CLU* knockdown affects VWF shunted into the “constitutive pathway” of VWF release, which is generally thought not to not involve WPB formation and primarily releases VWF into the basolateral surface [23]. A role for *CLU* that affects basolateral VWF secretion may be directionally consistent with other associated roles for *CLU* in angiogenesis, amyloidosis and neointimal hyperplasia [24–26], as these processes are more likely to affect the basolateral surface than the plasma surface of ECs. Other hypotheses to explain the dichotomy of similar WPBs but decreased intracellular/cellular supernatant VWF could include (1) an inability to resolve minor changes in WPBs (such as in multimeric structure) or (2) that *CLU* knockdown may alter the density of VWF packaging into WPBs and affect the overall “quanta” of VWF without dramatically affecting the total number of WPBs or the size of the WPBs. Further investigation into the polarity and WPB behavior (perhaps through real-time WPB assays and/or constitutive/basal/regulated pathways) after *CLU* knockdown may provide additional insight into this phenomenon. In a similar notion, further analysis of the multimeric structure of VWF may yield further information regarding the effects of *CLU* knockdown on VWF biology, specifically WPBs. Given the role (protein stabilization) and location (golgi) of *CLU*, it is reasonable to examine the effect that this may have on *VWF* multimerization.

The decreases of *VWF* at the protein level did not appear to be driven by transcriptional regulation of *VWF* by clusterin, as knockdown of *CLU* had no significant effect on *VWF* mRNA copy number. This result is consistent with the known biological functions of *CLU*, as there is little evidence of direct transcriptional activity of *CLU*, although it has been shown to affect certain pathways and gene sets via stabilization of transcriptional proteins [15]. The “cytoprotective” function of *CLU* may have functions in protecting VWF from pathways of autophagy or cellular recycling, and *CLU* knockdown/knockout may have decreased the total intracellular content of *VWF* but perhaps did not occur on a magnitude that was able to be resolved via WPB analysis. Alternatively, some of the organelles that we determined to be WPBs based on our imaging studies may have also been autophagic lysosomes that we targeted for degradation but still contained enough intact VWF to appear as WPBs in our imaging analysis. Further studies investigating co-staining for acidic lysosomes or for *LAMP1* may better delineate WPBs from lysosomes, although it is noted that WPBs are also acidic [27].

In the context of what is known regarding *CLU* in ECs, our data support the potential role of *CLU* in regulation of endothelial functions and VWF. As *CLU* has been associated with WPBs in HUVECs [17], it is not surprising that it may have some effect on VWF levels, although as noted there seem to be little effect on WPBs in our assays. Another report describes increases in multimeric size after *CLU* knockdown but does not further elucidate changes to overall VWF levels [22]. It is unclear what role an increased multimeric size would have on overall VWF levels and intracellular VWF levels. It is possible that alterations in multimeric size, may lead to changes in VWF levels such as that seen in ECFCs from Type 2 VWD patients [28].

An interesting theme in the role of *CLU* in ECs has been alterations in complement. Shear-stress induced upregulation of *CLU* decreased complement mediated EC activation [29] and in HUVECs, clusterin has been shown to regulate C5b-7 and C9 [30]. As VWF has also been shown to be involved in complement regulation [31, 32], it is possible that the roles of clusterin and VWF are linked by this pathway. As a protein stabilization protein, other pathways such as cellular recycling and autophagy-related mechanisms should also be considered. Additional experiments such as RNA-seq and/or proteomic analysis may identify cellular pathways.

From the perspective of correlation with the phenotype of VWD, we found that *CLU* knockdown decreases intracellular VWF content and/or VWF secretion. This would align with the clinical phenotype of VWD, which typically demonstrates decreased VWF plasma levels (akin to the cellular supernatant). However, decreased VWF secretion into the plasma (*in vivo*) or the supernatant (*in vitro*) is typically thought to occur via “regulated secretion” and “basal secretion,” both of which generally involve WPB pathways [33], and we did not find alterations in WPBs. As noted above, this dichotomy could be due to our inability to resolve minor changes in WPBs and/or effects on the “quanta density” of VWF in WPBs, or perhaps because *CLU* knockdown led to VWF-containing lysosomes that we misidentified as WPBs. Another possible hypothesis is that interactions of VWF and clusterin may occur extracellularly and correlate with the findings of Low VWF plasma levels in a clearance/stabilization-related manner such as Type 1C VWD. However, this hypothesis would not necessarily explain the decrease in intracellular VWF that we observe after *CLU* knockdown/knockout. As clusterin is also found in human plasma, further studies evaluating the binding of clusterin and VWF in plasma and/or cellular supernatant may reveal binding properties that affect the stabilization of VWF after cellular release.

Strengths of our report include a systematic evaluation of *CLU* on VWF biology, including evaluation at the transcriptional and the protein level. Additionally, as noted in the introduction, this investigation was sparked by an observation from ECFCs from patients with VWD, highlighting potential novel mechanisms that can lead to the clinical phenotype of VWD.

Limitations of our report include a lack of real-time visualization of WPB synthesis and WPB exocytosis. Similarly, we only evaluated WPBs by highlighting their VWF content, and did not evaluate other WPB cargo proteins, such as P-selectin [34, 35]. As it is unlikely that the effects of *CLU* would only affect VWF, it would be interesting to study the potential effects on other WPB cargo proteins. One experimental concern may be the differences in the time of incubation between our siRNA-treated cells and our *CLU*^*KO*^ cells. However, they displayed similar amounts of confluency and relatively similar WPB numbers and size. Additional limitations include using a pooled CRISPR-*CLU*^*KO*^, although we achieved significant knockdown of *CLU* expression (approximately 50%), it is likely that there were some non-knockout ECs in this pooled sample. Attempts to create a single cell colony clone of our CRISPR-*CLU*^*KO*^ cells were limited by cellular senescence of the transfected HUVECs. Future studies using iPSCs or HUVEC-hTERT cells as the target for *CLU* knockdown may be warranted.

In summary, we demonstrate that endothelial *CLU* knockdown resulted in decreased VWF levels, similar to findings in *ex vivo* ECs from patients with decreased VWF levels. This suggests that *CLU* may have a role in the regulation of VWF in ECs. Future studies should further evaluate potential mechanistic roles of *CLU* in VWF biology, perhaps with focuses on complement and WPB pathways.

## Data sharing

Single-cell RNA-sequencing data is available on dbGaP (phs002731.v1.p1) per the requirements of our IRB. Minimal dataset and uncropped western blots are available at: Ng, C. (2024). Clusterin knockdown has effects on intracellular and secreted von Willebrand factor in human umbilical vein endothelial cells ‐ DATA [Data set]. Zenodo. https://doi.org/10.5281/zenodo.10459109. This data will be released upon acceptance. Further data analysis, programming scripts, and raw data is available upon request. Please email Christopher.ng@cuanschutz.edu or allaura.cox@cuanschutz.edu for data requests. For a non-author contact, John Repine, john.repine@ucdenver.edu serves as the current Scientific Research Integrity Officer at CU-Anschutz.

## Supporting information

S1 File (DOCX)
