# Correction: Clusterin knockdown has effects on intracellular and secreted von Willebrand factor in human umbilical vein endothelial cells

**DOI:** 10.1371/journal.pone.0319850

**Published:** 2025-03-21

**Authors:** Allaura A. Cox, Alice Liu, Christopher J. Ng

The Data Availability statement for this paper is incorrect. The correct statement is: Single-cell RNA-sequencing data is available on dbGaP (phs002731.v1.p1) per the requirements of our IRB. Minimal dataset is available at: Ng, C. (2024). Clusterin knockdown has effects on intracellular and secreted von Willebrand factor in human umbilical vein endothelial cells - DATA [Data set]. Zenodo. https://doi.org/10.5281/zenodo.10452710. Further data analysis, programming scripts, and raw data is available upon request. Please email Christopher.ng@cuanschutz.edu or allaura.cox@cuanschutz.edu for data requests. For a non-author contact, John Repine, john.repine@ucdenver.edu serves as the current Scientific Research Integrity Officer at CU-Anschutz.
