# Supplementary material for: Clusterin knockdown has effects on intracellular and secreted von Willebrand factor in human umbilical vein endothelial cells
Source: PLoS One. 2024 Feb 16;19(2):e0298133. doi: 10.1371/journal.pone.0298133 (PMC10871512; doi:10.1371/journal.pone.0298133)
Supplement: S1 File — (DOCX) [file pone.0298133.s001.docx]

**Supplemental Material**

**S1 Table: List of reagents**

| **Reagent** | **Manufacturer** | **Location** | **Item Number** |
| --- | --- | --- | --- |
| Polyclonal Rabbit anti-human vWF antibody | DAKO | Carpinteria, CA | p0226/a0082 |
| Goat anti-rabbit HRP-linked secondary antibody | Cell Signaling Technology | Danvers, MA | 7074 |
| Albumin, Bovine, Fraction V, 97%, Standard Grade, pH 7.0 | ThermoFisher Scientific | Waltham, MA | J64655.22 |
| PBS | Gibco™ | Billings, MT | 10010023 |
| Triton™ X-100 Surfact-Amps™ Detergent Solution Triton-X | ThermoFisher ScientificSigma Aldrich | Waltham, MASt. Louis, MO | 85111 |
| 8% Paraformaldehyde (formaldehyde) aqueous solutionParaformaldehyde | Electron Microscopy SciencesSigma Aldrich | Hatfield, PASt. Louis, MO | 1578 |
| Phorbol-12-myristate-13-acetate (PMA) | Sigma Aldrich | St. Louis, MO |  |
| RIPA buffer | Cell Signaling Sigma Aldrich | Danvers, MA | 9806S |
| EGM-2 MV Microvascular Endothelial Cell Growth Medium-2 BulletKitTM | Lonza | Basel, Switzerland | CC-3202 |
| 0.25% Trypsin, 0.1% EDTA in HBSS w/o Calcium | Corning | Corning, NY | 25053CI |
| HyClone FBS defined (US) | Avantor | Radnor Township, PA | 16777-006 |
| 100 x Penicillin-Streptomycin Solution | Corning | Corning, NY | 30002CI |
| Opti-MEM™ I Reduced Serum Medium | Gibco™ | Billings, MT | 31985070 |
| Lipofectamine™ RNAiMAX Transfection Reagent | Invitrogen | Waltham, MA | 13778075 |
| Gelatin coated German coverslips | Neuvitro Corporation | Camas, WA | GG-12-15-Gelatin |
| Microscope Slides | Fisherbrand | Waltham, MA | 12-550-18 |
| ProLong™ Glass Antifade Mountant with NucBlue™ Stain | Invitrogen | Waltham, MA | P36983 |
| sheep anti-human VonWillibrand Factor: FTCI | Bio-Rad | Hercules, CA | AHP062F |
| Clusterin-α Antibody (B-5) antibody | Santa Cruz Biotechnology | Dallas, TX | sc-5289 |
| Goat anti-mouse IgG (H+L) Secondary Cy5 | Novus | Littleton, CO | NB7602 |
| AVW-1 | Versiti Blood Research Institute | Milwaukee, WI | n/a |
| 105.5 | Versiti Blood Research Institute | Milwaukee, WI | n/a |
| Calcium carbonate, 99+%, ACS reagent, Thermo Scientific Chemicals | Thermo Scientific Chemicals | Waltham, MA | 423515000 |
| Factor Assay Control Plasma | George King Biomedical | Overland Park, KS | 0020-0 |
| PBS | BioRad | Hercules, CA | 1610780 |
| Tween | Fisher Scientific | Waltham, MA | BP337-500 |
| H2SO4 | Fisher Scientific | Waltham, MA | S25899 |
| SIGMAFAST™ OPD | Sigma - Aldrich | St. Louis, MO | P9187-50SET |
| miRNeasy Mini Kit | Qiagen | Hilden, Germany | 217084 |
| Nuclease-Free Water (not DEPC-Treated) | Invitrogen | Waltham, MA | AM9937 |
| High-Capacity cDNA Reverse Transcription Kit | Applied Biosystems | Waltham, MA | 4374966 |
| Oligo d(T)16 (50 µM) | Invitrogen | Waltham, MA | N8080128 |
| PCR Tubes and Caps | Light Labs | Aurora, CO | A-4002-Z |
| TaqMan™ Universal PCR Master Mix | Applied Biosystems | Waltham, MA | 4304437 |
| MicroAmp™ Fast Optical 96-Well Reaction Plate with Barcode, 0.1 mL | Applied Biosystems | Waltham, MA | 4346906 |
| MicroAmp™ Optical Adhesive Film | Applied Biosystems | Waltham, MA | 4311971 |
| 10x Tris/Glycine Buffer | BioRad | Hercules, CA | 1610734 |
| Halt™ Protease Inhibitor Cocktail (100X) | Thermo Scientific | Waltham, MA | 78429 |
| 2x Laemmli Sample Buffer | BioRad | Hercules, CA | 1610737 |
| 2-Mercaptoethanol | Gibco | Billings, MT | 21985023 |
| 4–15% Mini-PROTEAN® TGX™ Precast Protein Gels, 10-well, 50 µl | BioRad | Hercules, CA | 4561084 |
| 10x Tris/Glycine/SDS Buffer | BioRad | Hercules, CA | 1610732 |
| Precision Plus Protein Dual Color Standards | BioRad | Hercules, CA | 1610374 |
| 10x Tris Buffered Saline | BioRad | Hercules, CA | 1706435 |
| Blotting-Grade Blocker | BioRad | Hercules, CA | 1706404 |
| Western Lightning ECL Pro | Revvity Health Sciences Inc | Waltham, MA | NEL120001EA |
| Qubit RNA BR Assay Kit | Invitrogen | Waltham, MA | Q10211 |

**S2 Table: List of siRNAs used**

| **Gene** | **ThermoFisher Scientific Assay ID** |
| --- | --- |
|  |  |
| **Control siRNA** | SIC001-10NMOL |
| ***CLU*** | s3157 |
| ***CLU*** | s3156 |
| ***CLU*** | s3158 |
| ***VWF*** | s14832 |
| ***VWF*** | s14833 |

**S3 Table: List of Taqman probes used**

| **Gene** | **Thermo-Fisher Item Number** |
| --- | --- |
| ***VWF*** | Hs01109446_m1 or Hs00169795_m1 |
| ***GAPDH*** | Hs02786624_g1 |
| ***CLU*** | Hs00156548_m1 |

**S4 Table: List of commercial cell lines used**

| **Cell Line** | **Manufacturer** | **Location** | **Catalog Number** |
| --- | --- | --- | --- |
| HUVEC | Lonza | Portsmouth, NH | C2517A |
